# The Impact of Sulfite Reduction Alternatives with Various Antioxidants on the Quality of Semi-Sweet Wines

**DOI:** 10.3390/foods15010053

**Published:** 2025-12-24

**Authors:** Zhenghai Liu, Ping Tang, Chenyu Wang, Shaosong Ye, Feng Xiao, Xueru Luo, Jun Wang, Jiang Lu, Wei Ji, Zhigang Dong, Qifeng Zhao

**Affiliations:** 1 College of Horticulture, Shanxi Agricultural University, Taiyuan 030801, China; lzh@sxau.edu.cn (Z.L.); 15982733518@163.com (P.T.); 18636931588@163.com (C.W.); yeshaosong152@163.com (S.Y.); xiaofeng2121055@163.com (F.X.); 2 Institute of Pomology, Shanxi Agricultural University, Taiyuan 030801, China; 3 School of Food Science and Engineering, Shanxi Agricultural University, Taiyuan 030801, China; 13466885298@163.com (X.L.); sxnyauwj@163.com (J.W.); 4 School of Agriculture and Biology, Shanghai Jiao Tong University, Shanghai 200240, China; jiang.lu@sjtu.edu.cn

**Keywords:** white wine, antioxidants, amino acids, volatile aroma compounds, sensory evaluation

## Abstract

This study is designed to identify strategies for substituting or reducing the use of sulfur dioxide (SO_2_) while improving the quality of semi-sweet wines. Using the ‘Petit Manseng’ semi-sweet wine as the research object, the effects of different concentrations and combinations of glutathione (GSH), mannan (Man), vitamin C (VC), chitosan oligosaccharide (COS), and SO_2_ were investigated. The results show that under VC treatment, the DPPH free radical scavenging rate of the wine is higher than that of other antioxidant treatments, with the V-1 treatment being 16.9% higher than the CK treatment. The wine treated with 30 mg/L of SO_2_ has the highest reducing power. After COS treatment, the wine color deepens, making it unsuitable for sweet white wines. GSH and Man can delay the increase in OD240 and the decrease in total phenolic. Man can increase the content of sweet amino acids such as serine. The treatments with man and GSH result in a greater variety of aromatic compounds and higher content of epigallocatechin. The combination treatment of GSH, Man, and SO_2_ resulted in semi-sweet wines with a bright color, rich aroma, and full-bodied, balanced taste, achieving the best quality indicators and the highest sensory evaluation. This wine exhibits a light yellow color, full body, and a complex, balanced aroma, which are improvements in quality compared to semi-sweet wines with 30 mg/L of SO_2_ added, and it reduces the use of SO_2_ by 33.3%. This study shows that adding 5 mg/L of GSH, 5 mg/L of Man, and 20 mg/L of SO_2_ can significantly improve the antioxidant properties and quality of semi-sweet wines.

## 1. Introduction

Sweet white wines are renowned for their high sugar content, low acidity, and complex aromas, but excessive oxidation severely affects their quality and stability. Oxidation in sweet white wines represents a long-standing technical challenge in the winemaking industry. It primarily involves the non-enzymatic reactions of phenolic compounds in the wine and oxygen, leading to color darkening and changes in aroma and flavor [1,2]. The chain reaction of oxidation often begins with the release of polyphenol oxidase and peroxidase from damaged cells, which catalyze the conversion of catechols to quinones in the presence of oxygen. These quinones further condense with amino acids and proteins to form brown polymers, causing the wine to take on an amber or even brownish hue [3]. Oxidation selectively affects the aroma of sweet wines, initially leading to the oxidative degradation of terpene compounds (such as geraniol and linalool), weakening the fruity and floral scents. Subsequently, aromatic alcohols such as benzyl alcohol and phenylethanol are converted into aldehydes, producing a nutty or sherry-like oxidized aroma [4]. More importantly, sulfur-containing amino acids decompose during oxidation to form thiols, which have an extremely low threshold (approximately 10 ng/L) and can impart an unpleasant rubber or rotten vegetable odor to the wine [5].

In modern winemaking processes, techniques to prevent oxidation mainly include physical barriers and chemical control. Inert gas protection (such as nitrogen or argon) effectively reduces the contact between wine and oxygen [6], while the addition of sulfites (30–50 mg/L free SO_2_) inhibits oxidative enzyme activity by scavenging free radicals. However, free SO_2_ can combine with terpene aroma compounds (such as nerol), weakening the fruity and floral scents [7]. Wines with the addition of free SO_2_ pose certain health risks to the human body. Excessive SO_2_ in the body must be converted into sulfate by sulfite oxidase and then excreted. Long-term intake may increase the metabolic burden on the liver, especially for those with impaired liver function [8] and may also trigger asthma attacks, rashes, or breathing difficulties. The U.S. FDA (United States Food and Drug Administration) [9] requires all foods containing more than 10 mg/L of SO_2_ to be labeled with a warning, and the World Health Organization [10] and the International Organization of Vine and Wine [11] have established regulatory restrictions on the addition of sulfites. Moreover, winemakers are also concerned that excessive use of SO_2_ in grape juice and wine can negatively affect wine quality and produce unpleasant flavors. Therefore, there is an urgent need to identify new antioxidants to replace or reduce the addition of SO_2_ to wine. Studies have shown that VC (Vitamin C), GSH (Glutathione), and Man (Mannan) can serve as antioxidants in wine, potentially replacing SO_2_. OIV Resolution OIV-OENO 445-2015, “Treatment of Must with Glutathione,” is an important technical specification released by the International OIV in 2015. The core content permits the addition of GSH in must as an antioxidant to reduce the use of sulfur dioxide (SO_2_). Man (Mannan), as a natural component, is used in accordance with the relevant regulations of the OIV. VC consumes oxygen through its own oxidation, thereby reducing the oxidation of wine [12]. The Universitat Rovira i Virgili used Muscat of Alexandria grape juice to compare fermentation with the addition of 20 mg/L of GSH and 20 mg/L of sulfur dioxide (SO_2_). The results show that GSH can effectively prevent the browning of white grape juice. [13].

Phenolic compounds extracted from grape pomace as by-products show increased scavenging rates of DPPH (1,1-Diphenyl-2-picrylhydrazyl) free radicals with higher concentrations. However, excessive concentrations can deepen the wine’s color and enhance astringency. This occurs because phenolic compounds combine with proteins and metal ions in the wine to form large molecular complexes. These complexes alter the intermolecular interactions in the wine, affecting how the wine interacts with the oral mucosa and thereby intensifying the astringent sensation. Moreover, the formation of the complexes has changed the light absorption and scattering properties of the substances in the wine, giving the wine a deeper color [14].

In recent years, consumers have increasingly favored semi-sweet wines, and many domestic and international studies have been conducted to improve the quality of semi-sweet wines. However, few studies have investigated the effects of adding antioxidants after bottling on the quality of semi-sweet wines [15,16]. In light of the health issues associated with SO_2_ addition, it is urgent to clarify the effects of adding natural antioxidants on wine quality during bottle storage. ‘Petit Manseng’ is a late-ripening, thick-skinned, and loosely clustered grape variety that has high sugar content and moderate acidity. Its distinctive aromas include ripe peach, honey, and cinnamon, making it well-suited for producing high-quality sweet and semi-sweet wines [17,18]. Adding 30 mg/L of GSH before grape pressing can significantly enhance the antioxidant capacity and aroma stability of Sauvignon Blanc wine [19]. A dosage of 40 mg/L of GSH notably improves the bitterness and aroma intensity of white wine [20]. Man can protect phenolic compounds in red wine and promote the formation of pyranoanthocyanins, regulating and retaining the content of effective aromatic compounds [21]. The use of 40–250 mg/L of VC at white wine bottling can impact the aroma, taste, and clarity of the wine [12]. COS (Chitosan oligosaccharide), a natural polymer, has good antibacterial, chelating, clarifying, and antioxidant activities [22]. COS treatment during the veraison and maturation of Cabernet Franc grapes can significantly increase the levels of total phenols, total flavonoids, and total anthocyanins in grape berries, with noticeable effects on flavanols, phenolic acids, flavonols, and stilbenes [23]. These four natural antioxidants may serve as green alternatives to sulfites in wine.

While current wine antioxidant research predominantly focuses on single compounds or traditional antioxidants, this study presents a novel approach by innovatively combining four antioxidants with diverse functions, namely GSH, Man, VC, and COS, and systematically analyzes their impacts on the quality of semi-sweet white wines during bottle storage. A multi-dimensional analysis approach is employed, comprehensively covering key aspects such as antioxidant indicators, color indicators, phenolic substances, amino acid content, volatile aroma components, and sensory quality. This study thoroughly investigates the distinct mechanisms by which different antioxidants and their combinations influence wine quality, accurately determines the optimal antioxidant combination and dosage, and provides a novel solution with both theoretical significance and practical value for improving the quality of semi-sweet wines. This study contributes to the understanding of the synergistic effects of multiple antioxidants in sweet wines and provides a new pathway to enhance the quality of the wine industry.

## 2. Materials and Methods

### 2.1. Winemaking Materials and Conditions

The grape variety used in the experiment is ‘Petit Manseng’, cultivated in the wine grape variety garden of the Institute of Pomology, Shanxi Agricultural University (N37.34°, E112.49°, altitude 846 ± 5 m). The grapes were planted in 2011 using a single dragon vine “factory” trellis system, with standard vineyard management practices applied throughout the year. The grapes were harvested for semi-sweet wine production when the soluble solids content exceeded 25% (*w*/*w*). The winemaking process was as follows: Five hundred kilograms of harvested grapes were transported to the experimental winemaking facility and gently pressed, with 50 ppm of sulfurous acid added during the pressing process. Then, the grape juice was separated from the grape skins and seeds. The freshly pressed grape juice was left standing in an environment of 0 °C for 48 h. Then, the clarified grape juice on the upper layer was transferred to a 200 L fermentation tank using siphoning. The tank was filled with an appropriate amount of Angel BV818 yeast at a concentration of 0.2 g/L for fermentation. The fermentation temperature was maintained between 13 °C and 16 °C. When the specific gravity reached 0.998–0.999, the fermentation process was terminated using a low-temperature method. The physical and chemical indexes of the wine are shown in Table 1. The wine was canned according to the experimental setup. The wine was bottled in 375 mL white Bordeaux bottles, with 30 bottles prepared for each treatment. The bottled wine was stored in a constant temperature basement at 15 °C. The Single treatment and Combined treatment used different batches of wine, and the physicochemical indexes of the two batches of wine were slightly different.

### 2.2. Experimental Treatments

**Figure 1:**
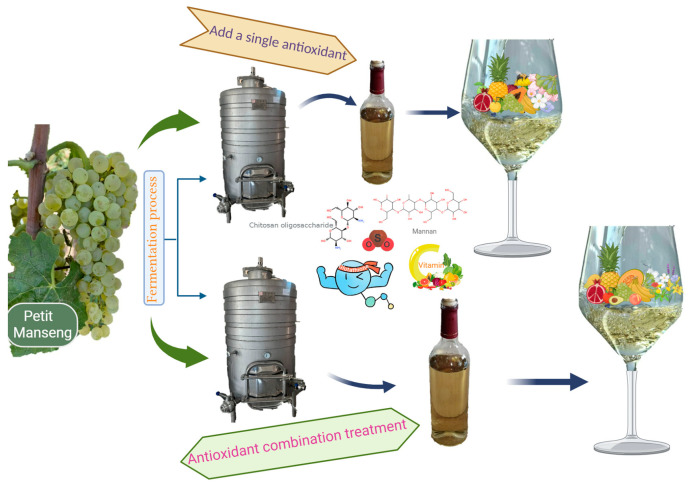
Experimental treatment schematic diagram.

The experimental treatments are shown in Figure 1. Antioxidants at different concentrations were added to the wine at the time of bottling. The different antioxidants were purchased from Beijing Solarbio Science & Technology Co., Ltd. (Beijing, China), and the VC used was L-ascorbic acid. Man was formed by the covalent bonding of 80% to 90% mannose and 5% to 20% protein. The antioxidants were added in the form of solutions. The antioxidant treatment methods are shown in Table 2: ① Blank control without antioxidant (CK); ② GSH added at 10, 20, and 30 mg/L (termed G-1, G-2, and G-3, respectively); ③ Man added at 10, 20, and 30 mg/L (termed M-1, M-2, and M-3, respectively); ④ VC added at 50, 100, and 200 mg/L (termed V-1, V-2, and V-3, respectively); ⑤ COS added at 50, 100, and 200 mg/L (termed C-1, C-2, and C-3, respectively); ⑥ SO_2_ added at 10, 20, and 30 mg/L (termed S-1, S-2, and S-3, respectively). Antioxidants used in single treatments and combined treatments were not from the same batch of wine. The combined antioxidant treatments were as follows: blank control without antioxidant (CK*); GSH added at 10 mg/L (G*); Man added at 10 mg/L (M*); both GSH and Man added at 10 mg/L each (GM*); SO_2_ added at 20 mg/L (S*); GSH added at 10 mg/L and SO_2_ added at 20 mg/L (GS*); Man added at 10 mg/L and SO_2_ added at 20 mg/L (MS*); both GSH and Man added at 5 mg/L each and SO_2_ added at 20 mg/L (GMS*). After six months of bottle storage, the various indicators were measured.

### 2.3. Analyses of Wines After the Addition of Antioxidants

#### 2.3.1. Determination of DPPH Radical Scavenging Activity

The method of Kmi et al. [24] had been referenced and slightly modified. 2.8 mL of the diluted wine sample (diluted 10 times) had been added to the methanol solution containing 0.2 mmol/L DPPH. After obtaining a reaction system with a volume of 3 mL, it had been shaken until evenly mixed. After mixing by shaking, the system had been kept in the dark at 25 °C for 0.5 h. Then, the absorbance had been measured at a wavelength of 517 nm. DPPH calculation formula is as follows:

DPPH radical scavenging rate (%) = $(1-\frac{A_{i}-A_{j}}{A_{0}})\times 100\%$

*A*_0_: The absorbance of the blank control group (that is, the methanol solution containing only DPPH and without the wine sample to be tested).

*A_i_*: The absorbance of the reaction system after adding the wine sample to be tested.

*A_j_*: The absorbance of the wine sample to be tested itself at a wavelength of 517 nm.

An ultraviolet spectrophotometer (model number T2600; Shanghai Youke Instrument Co., Ltd., Shanghai, China) was used for the analysis.

#### 2.3.2. Determination of Hydroxyl Radical Scavenging Activity

Based on the method of Liu et al. [25], diluted wine sample to be tested were mixed with FeSO_4_ solution and salicylic acid solution. Then, H_2_O_2_ solution was added to obtain a 6 mL reaction system. After mixing by shaking, the system was kept in a water bath at 37 °C for 1 h. Then, the absorbance was measured at a wavelength of 510 nm.

An ultraviolet spectrophotometer (model number T2600; Shanghai Youke Instrument Co., Ltd.) was used for the analysis.

#### 2.3.3. Determination of Wine Color

Due to the dark polymers produced by oxidation, the color of semi-sweet wine is an important indicator to determine the degree of oxidation, which can be used to judge the oxidation degree and quality of a bottle of wine. The spectrophotometric value at a wavelength of 420 nm was measured with a spectrophotometer [26].

#### 2.3.4. Determination of Total Phenols and Total Flavonoids

The determination of total phenols had been performed using the Folin–Ciocalteu method. Here, 0.5 mL of the wine sample had been added to a graduated test tube along with 1 mL of Folin–Ciocalteu reagent, 2.5 mL of 75 g/L Na_2_CO_3_ solution, and 2.3 mL of distilled water. A stopper had been placed on the tube. The solution had been shaken and then incubated in a water bath at 30 °C in the dark for 40 min, and then the absorbance had been measured at 762 nm. The structure of tannic acid has similarities with many phenolic substances in grapes, all of which contain multiple phenolic hydroxyl groups. These phenolic hydroxyl groups are the key structures for phenolic substances to exert various chemical properties and biological activities. The content of phenolic compounds had been calculated according to the standard curve of tannic acid (0–60 mg/L). Total flavonoids had been determined using the aluminum chloride colorimetric method. Briefly, 0.5 mL of the wine sample had been mixed with 0.3 mL of 5 g/100 mL NaNO_2_ solution. After 5 min, 0.3 mL of 10 g/100 mL AlCl_3_·6H_2_O solution had been added and mixed well. After another 5 min, 2 mL of 1 mol/L NaOH had been added. After the reaction solution had been fully mixed and kept in the dark for 15 min, the absorbance had been measured at a wavelength of 510 nm. The content of total flavonoids had been calculated according to the standard curve of catechin (0–100 mg/L) [27]. An ultraviolet spectrophotometer (model number T2600; Shanghai Youke Instrument Co., Ltd.) had been used for the analysis.

#### 2.3.5. Determination of Monomeric Phenols

Monomeric phenols were determined using high-performance liquid chromatography. The samples were filtered through a 0.45 μm microporous membrane before assessment. The HPLC (High-Performance Liquid Chromatography) conditions were as follows. An Inertsil ODS-C18 column (250 mm × 4.6 mm × 5 μm) was used for measurement based on the following conditions: column temperature, 30 °C; injection volume, 20 μL; UV detection wavelength, 280 nm; flow rate, 1 mL/min; and maximum pressure limit, 300 bar. The quantification was performed using the external standard method based on the peak area. The mobile phase A was 2% acetic acid, and the mobile phase B was 100% acetonitrile. Gradient elution was used with the following elution program 0 min, 86% A and 14% B; 0~8 min, 95% A and 5% B; 8~15 min, 86% A and 14% B; 15~25 min, 60% A and 40% B; and 25~30 min, 86% A and 14% B.

The measuring equipment used is a high-performance liquid chromatograph (model number Agilent 2500; Agilent Technologies Co., Ltd., Santa Clara, CA, USA) [28].

#### 2.3.6. Determination of Amino Acids

The chromatographic column was AdvanceBio AAA (4.6 × 100 mm, 2.7 μm), and the column temperature was 40 °C. The mobile phase A consisted of 10 mmol/L sodium hydrogen phosphate and 10 mmol/L sodium tetraborate, with a pH of 8.2; the mobile phase B was methanol/acetonitrile/pure water = 45:45:10 (*v*/*v*/*v*). The elution program was as follows: 0–0.35 min, 2% B; 0.35–13.4 min, 2–57% B; 13.4–13.5 min, 57–100% B; 13.5–15.7 min, 100% B; 15.7–15.8 min, 100–2% B; and 15.8–18 min, 2% B. The flow rate was 1.5 mL/min. The following fluorescence detection wavelengths were used: 1–11.4 min, λex = 340 nm, λem = 450 nm; 11.5–18 min, λex = 230 nm, λem = 305 nm. The injection volume was 5 μL.

For the online derivatization injection procedure, 2.5 μL of boric acid buffer was drawn, followed by 2.0 μL of sample. The mixture was mixed twice at the maximum speed and kept for 0.5 min. Then, 0.5 μL of OPA (o-phthalaldehyde) was drawn and mixed six times at the maximum speed. After that, 0.5 μL of FMOC (9-fluorenylmethyloxycarbonyl) was drawn and mixed six times at the maximum speed. Finally, 32 μL of pure water was drawn and mixed twice at the maximum speed before injection.

Based on the retention time of the standard substances and the standard curve, qualitative and quantitative analyses were performed using the chromatographic analysis results of the mixed amino acid standards. The standard substances were of chromatographic purity and were purchased from Shanghai Yuanye Biotechnology Co., Ltd. (Shanghai, China).

#### 2.3.7. Determination of Volatile Compounds

The determination of volatile compounds in wine was performed using headspace solid-phase microextraction.

For preparation of the internal standard solution, 2-octanol was diluted 100 times with ultrapure water to a final concentration of 12 μL/100 mL, mixed well, and stored at 4 °C. The 2-octanol standard substance was of chromatographic purity and was purchased from Shanghai Yuanye Biotechnology Co., Ltd.

For sample preparation, 8 mL of the wine sample was added to a 20 mL headspace vial, with 0.3 g/mL NaCl. The sample was preheated at 45 °C for 10 min, adsorbed for 30 min, and then analyzed using GC (Gas Chromatography) for 5 min.

The following chromatographic conditions were employed: TR-5MS column (30 m × 0.25 mm ID × 0.25 um film); the initial temperature was kept at 40 °C for 2 min, then increased to 130 °C at a rate of 3 °C/min, and finally increased to 230 °C at a rate of 3 °C/min and kept for 2 min; the injection was performed without splitting; the injection port temperature was 260 °C; and the carrier gas flow rate was 1 mL/min.

Regarding spectrometry conditions, the ion transfer line temperature was 250 °C, the ion source temperature was 250 °C, the ionization mode was EI, the electron energy was 70 eV, and the scanning mass range was 29~350.

Both qualitative and quantitative methods were employed for aroma compound analysis. Qualitative identification was performed by comparing mass spectrometry with a reference database, retention times, and the relevant literature. Quantitative analysis was performed using the internal standard method, in which the concentration of each aroma compound was represented by the ratio of the peak area of each substance in the tested sample to the peak area of the internal standard (2-octanol) multiplied by the concentration of the internal standard [28].

A GC-MS (Gas Chromatography-Mass Spectrometry) gas chromatograph (model number Trace1300; Thermo, Waltham, MA, USA) was used to obtain the measurements.

### 2.4. Sensory Evaluation Method

According to the national standard GB15038 [29], a 10-person evaluation group was formed. The members of the evaluation group were composed of students and teachers from Shanxi Agricultural University majoring in wine. These students were required to have no basic taste and olfactory deficiency and a good sense of smell. They previously underwent training in wine nose memory and aroma description. Among the panel members, the distribution by birth year and gender was as follows:1 male and 1 female born after 2000; 2 males and 1 female born between 1990 and 1999; 1 male and 2 females born between 1980 and 1989; 1 female born between 1970 and 1979; 1 male born after 1969. Among the members of the expert group, the distribution by year of birth and gender is shown in Table 3:

**Table 3:** Tasting Panel Age Composition Table.

Birth Year	Number and Gender
born after 2000	1 male and 1 female
born between 1990 and 1999	2 males and 1 female
born between 1980 and 1989	1 male and 2 females
born between 1970 and 1979	1 female
born after 1969	1 male

The sensory evaluation experiment was performed in the wine tasting room. Each wine sample was evaluated twice, and 30 mL of wine sample was poured into a standard wine glass. The wine samples were randomly numbered and sorted. The sensory evaluation was scored as follows: 20 points for visual assessment, with 10 points each for color and clarity; 30 points for olfactory assessment, with 15 points each for aroma types and intensity; and 50 points for taste assessment, with 20 points each for body balance and flavor expression and 10 points for no defects. The comprehensive scores were used to compare the sensory differences in semi-sweet wines after different treatments. Ethical approval does not apply to this article. Sensory evaluation participants provided informed consent to participate in the sensory study and agreed to use their data for research purposes [28].

### 2.5. Statistical Analysis

Each treatment was repeated three times, and the final results were expressed as the mean values. The data obtained from the experiments were processed using the statistical software IBM SPSS Statistics version 19, and graphs were created using Excel software, the Hiplot scientific plotting platform (https://hiplot.com.cn/), and SIMCA software (Version 14.1).

## 3. Results and Discussion

### 3.1. Effects of Different Antioxidants on Semi-Sweet Wines

#### 3.1.1. Effects of Different Antioxidants on the Antioxidant Capacity of White Wines

**Figure 2:**
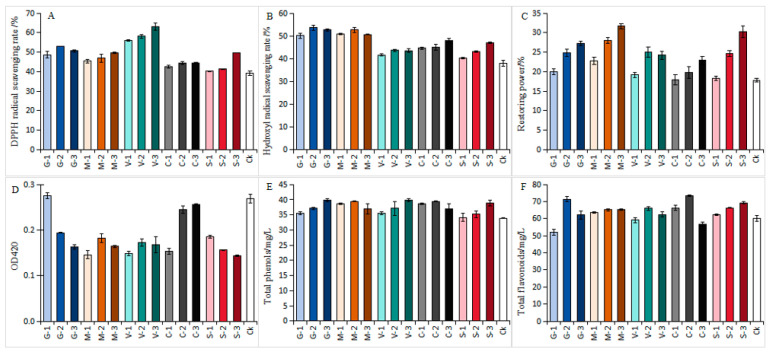
(**A**–**F**) Differences in the antioxidant capacity of white wines after treatment with different concentrations of antioxidants. (**A**) The DPPH radical scavenging rate, (**B**) the hydroxyl radical scavenging rates, (**C**) the reducing power, (**D**) the OD420 values, (**E**) the total phenol content, and (**F**) the total flavonoid content in white wines after treatment with different concentrations of antioxidants.

Bottled white wines are particularly sensitive to dissolved oxygen, making them highly susceptible to non-enzymatic browning during storage. Unlike red wines, which have a darker color, white wines have a clear appearance that is more easily affected by browning [30]. Figure 2 presents the impact of various concentrations of antioxidants on the antioxidant properties of ‘Petit Manseng’ wine after six months of bottle storage. The DPPH radical, a widely used indicator, was used to assess the antioxidant capacity of different wine samples. After six months of storage, the DPPH radical scavenging rate of the bottled white wine was higher than the control for all antioxidant treatments (Figure 2A). The DPPH scavenging rate of the semi-sweet white wine with VC treatment was higher than that of other antioxidant treatments. The DPPH scavenging rate of the samples treated with high concentrations of SO_2_ was notably higher than the CK, and the DPPH scavenging rate obtained with GSH treatment was higher than that of Man treatment at the same concentration. Piergiorgio Comuzzo et al. [31] also obtained similar results for white wine, demonstrating that VC was very effective in scavenging oxygen and free radicals.

Hydroxyl radicals, important reactive oxygen species in the body with a strong ability to gain electrons (oxidize), induce numerous pathological changes, and the hydroxyl radical scavenging rate in wine samples reflects the effectiveness of antioxidant action. Figure 2B shows differences in the hydroxyl radical scavenging rates of the wines treated with different antioxidants. The hydroxyl radical scavenging rates of the wine samples treated with different concentrations of GSH and Man were higher than those treated with SO_2_ and the control. Specifically, semi-sweet wine treated with 20 mg/L GSH and Man had higher hydroxyl radical scavenging rates of 53.7% and 52.7%, respectively. These values are 6.74% and 5.7% higher than those of S-3.

Figure 2C shows the reducing power of semi-sweet wine after six months of storage with different antioxidant treatments. High concentrations of antioxidants increased the reducing power of semi-sweet white wine. Here, the sample treated with 30 mg/L SO_2_ had the highest reducing power, and the reducing power of wine treated with 20 mg/L Man was greater than that of the control.

OD420 was used to measure the color of white wine, representing an important quantitative indicator for assessing the color depth of white wine. Figure 2D shows the OD420 values of semi-sweet wine after six months of bottle storage with different antioxidant treatments. As the concentration of GSH treatment increased, the OD420 value decreased, while the OD420 value increased as the concentration of the COS treatment agent increased. COS reacts with glucose in semi-sweet wine through the Maillard reaction to form brown substances, which is why COS is not suitable for sweet wines [32]. GSH, Man, and VC improve color parameters [33], but high concentrations of SO_2_ treatment could reduce the OD420 value.

Phenolic and flavonoid compounds, which are important antioxidants, potentially slow down the oxidation process of wine, helping to maintain the color, aroma, and flavor of wine. Figure 2E,F show the total phenolic and flavonoid content of semi-sweet wine after six months of bottle storage with different antioxidant treatments. The treatment agents delay the oxidation of total phenolics and flavonoids in wine. The total phenolic and flavonoid content in the wine samples increased as the SO_2_ concentration increased. Phenolic compounds in white wine were positively correlated with the hydroxyl radical scavenging rate [34,35]. In different antioxidant treatments, the total phenolic content in semi-sweet wine treated with 20 mg/L GSH and Man was higher, increasing by 9.3% and 13.9% compared to the control, respectively. Moreover, after treatment with G-2, the total phenol content in the wine was only 7.1% and 6.9% lower than that treated with G-3 and S-3, respectively. After treatment with C-2, the total flavonoid content in the wine was relatively high.

#### 3.1.2. Sensory Evaluation of Semi-Sweet Wine Under Different Antioxidants

**Figure 3:**
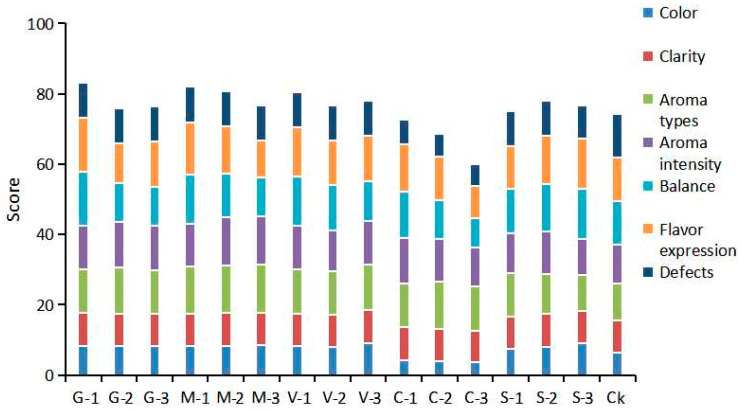
Sensory descriptive scores of semi-sweet white wine treated with different concentrations of antioxidants.

Sensory evaluations were conducted on semi-sweet wines treated with different antioxidants, and the scoring results are shown in Figure 3. The wines treated with GSH, Man, and VC received higher scores than the control. GSH, known as a potent antioxidant, preferentially reacts with oxygen or other oxidizing agents in the wine, reducing the proportion of phenolic substances in the grapes that were oxidized [36]. The wines treated with GSH were superior to the control group in terms of body balance. After treatment with Man (M-1), the color was maintained to a lighter shade, and the aroma intensity and body complexity were better than those of the blank control group. Man physically or chemically interacts with phenolic substances in white grapes through hydrogen bonds or other means, preventing the polymerization reactions between phenolic substances and color changes induced by polymerization [37]. This binding potentially alters the optical properties of phenolic substances, making the wine visually more stable and brighter in color [38]. The addition of VC increased the balance of the wine, with no outstanding performance in terms of aroma intensity and flavor. Here, VC potentially reduced the oxidation and polymerization of phenolic substances into quinones [39], thus preventing the wine from developing a darker tone and maintaining the originally lighter color of white wine, such as pale yellow or yellow–green. Because the wine samples treated with COS showed a deepening of color, they had obvious defects. The Aroma types and Aroma intensity scores of the wine samples treated with different concentrations of GSH and Man were higher than those of the wine samples with added SO_2_. This might be because the addition of SO_2_ weakened the aroma of the wine. The color score of the S-3 wine sample was the highest. SO_2_ treatment made the wine more pronounced, and high concentrations of SO_2_ treatment yield a sulfurous odor. The wine treated with 20 mg/L SO_2_ had relatively higher scores compared to the other two SO_2_ treatments. Sensory evaluations suggested that GSH and Man improved the sensory characteristics of the wine, whereas treatment with COS was inferior to the control group in many aspects, indicating that it was not suitable as an antioxidant treatment of semi-sweet wine.

### 3.2. Treatment of Semi-Sweet White Wine with Combinations of Various Antioxidants

The impact of various antioxidants on the quality of semi-sweet wine was unique. SO_2_ played a significant role in inhibiting spoilage microorganisms and ensuring wine quality, but high concentrations were required for noticeable antioxidant effects. However, high SO_2_ concentrations could affect the taste and pose health risks. To reduce the dosage of SO_2_, combination treatment with natural antioxidants Man and GSH effectively reduced the activity of spoilage microorganisms, decreased the amount of SO_2_ used, and significantly improved the quality of the wine [40]. Studies on white wine indicated [41] that combined VC and SO_2_ treatment accelerates the consumption of SO_2_ and enhances the production of yellow pigments, making it unsuitable for use in conjunction with SO_2_ in the treatment of white wine.

#### 3.2.1. The Combined Effects of Various Antioxidants on the Polyphenol Content in Semi-Sweet Wine

Phenolic compounds in wine can neutralize free radicals produced by the human body [42]. The variety and content of phenolic compounds and amino acids in wine, as well as their combinations, can lead to differences in the wine’s antioxidant capacity. The synergistic action of iron and copper ions with oxygen accelerates the photodegradation of methionine to produce methional, whereas monophenolic substances (such as caffeic acid) inhibit this reaction [43]. As shown in Table 4, differences in the content of phenolic substances were noted in semi-sweet wine treated with various combinations of antioxidants. Significance analysis between different treatments was conducted through analysis of variance (ANOVA). When GSH, Man, and SO_2_ were added separately, the content of Gallic acid, Epigallocatechin, Caffeic acid, Coumaric acid, and other substances in the wine was significantly lower than that in the wine samples treated with natural antioxidants combined with SO_2_. The combination treatment could significantly increase the content of individual phenols in the wine. Among the 18 types of monophenols detected, 11 were found at higher levels in the GMS* treated wine compared with the other treatments. Specifically, catechin, epicatechin, and gallic acid were present at levels 463.5%, 583.3%, and 767.9% greater than that noted in CK*, respectively. Additionally, the astringent acylated epicatechin gallate, which affects taste, was found at higher levels in the CK* and S* treatments, indicating that natural antioxidants can protect high-quality tannins and degrade low-quality tannins. The content of caffeic acid in the GMS*-treated wine was 250.4% higher compared with CK*. GS*- and GMS*-treated wines had higher caffeic acid content compared with other treatments, demonstrating that the antioxidant GSH protects caffeic acid from oxidation. Caffeic acid can delay the reduction in non-anthocyanin phenolic substances such as flavan-3-ols, flavonols, and phenolic acids [44].

#### 3.2.2. The Combined Effects of Different Antioxidants on the Amino Acid Content in Semi-Sweet Wine

Amino acids can undergo the Maillard reaction with reducing sugars in wine, producing a variety of compounds with roasted and toasted flavors, adding unique flavor layers to the wine and enriching its taste and complexity [45]. Some amino acids also possess certain viscous and colloidal properties, which can increase the body thickness and fullness of the wine. Amino acids play a role in the perception of taste and aroma in wine [46]. As shown in Table 5, under the combined effects of different antioxidants, the amino acid content in semi-sweet wine varied. In the samples treated with Man antioxidants, the content of sweet amino acids such as serine, glycine, and asparagine was increased compared with the control, with glycine’s sweetness being approximately 0.8 times that of sucrose [47]. Proline had the highest content in semi-sweet wines. The proline content in the M* wine sample was 2671.1 mg/L, which was 112.5 mg/L higher than that in the S* wine sample. Moreover, the total amino acid content in the M* wine sample was higher than that in the S* wine sample. Under GMS* treatment, the proline content in the wine sample was 86.3 µg/L higher than that in the CK* wine sample. The special cyclic structure of proline facilitated its combination with specific areas of taste receptors in the mouth, triggering corresponding neural signal transmission, thus allowing people to perceive sweetness. The sweet characteristics of proline impact the flavor of the product [48]. Asparagine, tyrosine, and ornithine were found at higher concentrations in samples treated with M* and MS*. This may be due to the addition of Man antioxidants during storage slowing down oxidation or increasing the content of these amino acids, thereby increasing the levels of ester and alcohol substances in the wine and enriching its aromatic compounds. The formation of fruity flavors and ester compounds in wine is closely associated with branched-chain amino acids, which can be converted into higher alcohols under certain conditions and subsequently transformed into ester substances. The amino acid composition is related to grape varieties, and these amino acids can be converted into specific aroma substances, thus forming different styles of wines [49]. The addition of different antioxidants exhibits correlation with the amino acid content in the wine. Procopio et al. [50] also confirmed that valine, glutamic acid, proline, leucine, and isoleucine were the most important explanatory variables affecting the content of aromatically active components, and a linear relationship was noted between the amino acid content and volatile aroma components under wine fermentation conditions.

#### 3.2.3. PCA of Monophenols and Amino Acids in Semi-Sweet Wine Under the Combined Effects of Different Antioxidants

**Figure 4:**
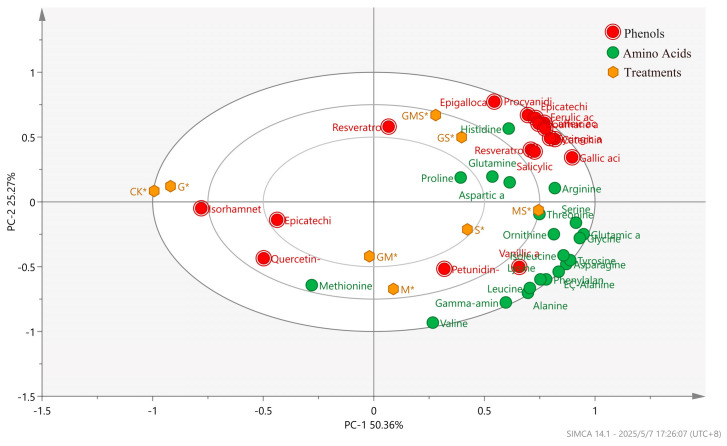
PCA of monophenols and amino acids in semi-sweet wine under the combined effects of various antioxidants.

As shown in Figure 4, the PCA diagram of monophenols and amino acids in semi-sweet wine under the combined effects of various antioxidants indicates that the horizontal axis, representing the first principal component, explained 50.3% of the total variance, while the vertical axis, representing the second principal component, accounted for 25.2% of the variance. Together, the two principal components explained 75.6% of the variance, demonstrating significant differences in monophenols and amino acids in semi-sweet wine after combined treatment with different antioxidants. The control group (CK) had the lowest loading values, indicating that the combined treatment with various antioxidants significantly enhanced the levels of monophenols and amino acids in semi-sweet wine. The MS* and GM* treatments had higher scores on the first and second principal components, respectively. The GS* treatment showed a strong correlation with amino acids such as threonine, serine, arginine, and glutamic acid, while the MS* treatment had a strong correlation with monophenols like resveratrol 3-O-glucoside, epicatechin, and resveratrol. This suggests that GSH and Man have a unique impact on the levels of monophenols and amino acids in semi-sweet wine, with Man being able to increase the retention rate of monophenols. The GMS treatment had higher loading values, associated with a variety of monophenols and amino acids, thereby creating an enriched and complicated wine flavor.

#### 3.2.4. Analysis of the Differences in Aroma Compounds in Semi-Sweet Wine Treated with Various Antioxidants

**Figure 5:**
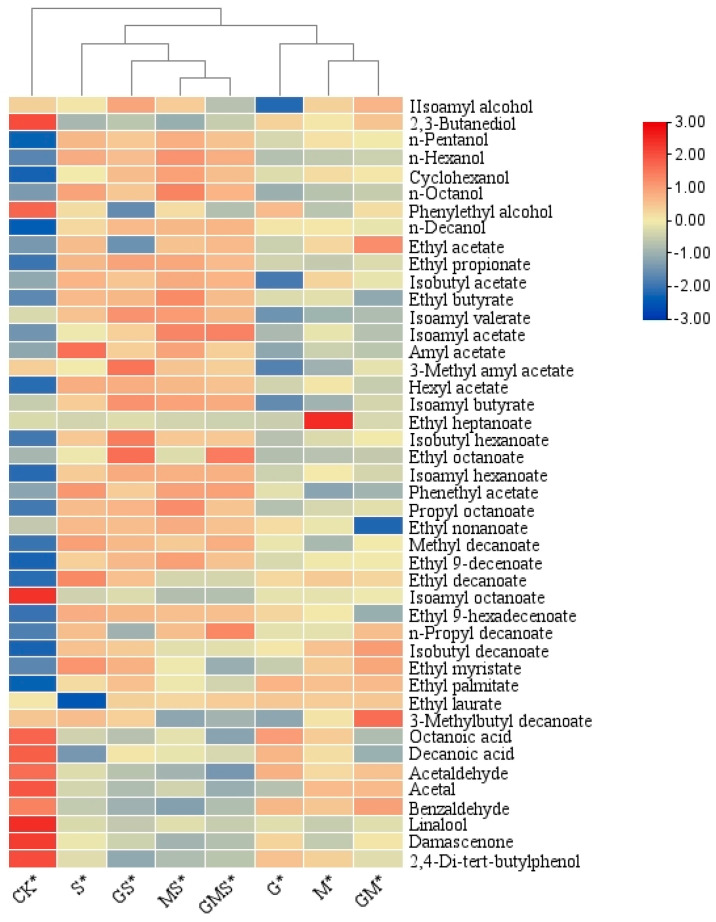
Heatmap of aroma compounds in semi-sweet white wine under the combined effects of various antioxidants.

SPME-GC/MS (Solid-Phase Microextraction Gas Chromatography/Mass Spectrometry) analysis was conducted to examine the differences in aroma compounds in semi-sweet white wines after six months of bottle storage following combined treatments with various antioxidants. As shown in Table 6, significant differences in the types and concentrations of volatile chemical substances were noted in the wines subject to different treatments. Esters and alcohols were the primary aroma components in ‘Petit Manseng’ semi-sweet wine, providing rich fruity and fatty aromas [51]. Alcoholic aroma compounds refer to organic compounds containing hydroxyl (-OH) functional groups, which possess a certain degree of volatility. As shown in Figure 5, the heatmap of volatile chemical substances in wine samples after different treatments indicates that the contents of acids such as Octanoic acid and Decanoic acid, and aldehydes such as Acetal and Acetaldehyde are the highest in CK. This may be related to the oxidation of the wine samples. Among the eight groups of wine samples, the highest content of alcohol volatile substances was found in MS* and GS*. The wines treated with GSH and Man exhibited higher concentrations of volatile compounds, mainly esters and alcohols, compared to those without natural antioxidant treatments. Regarding ester aroma compounds, the wine samples treated with GS* had higher contents of these compounds, which endowed the wines with a complex fruity and floral bouquet. This characteristic was significantly different from that of wine samples treated with other compounds. The contents of Isobutyl decanoate and 3-Methylbutyl decanoate, which have a fruity aroma, were significantly higher in the GS* wine samples than in the S* wine samples. The blank control group had higher contents of acidic compounds, phenethyl alcohol, and other alcohol- and high-acid-containing substances, with octanoic acid exhibiting the highest concentration. The combined effects of various antioxidants greatly improved the flavor of ‘Petit Manseng’ semi-sweet wine. GSH had a regulatory effect on the types and content of aromatic substances in the wine [52]. The wine samples treated with GSH had higher contents of ester compounds. Specifically, the total ester content in the wine samples treated with GMS increased by 71.35% compared to the CK*. This suggests that GSH may promote the formation of ester compounds. Moreover, six types of ester aromatics, including isopentyl acetate, hexyl acetate, isopentyl butyrate, octanoic methyl ester, ethyl 9-hexadecenoate, n-propyl decanoate, and ethyl laurate, reached their highest concentrations in the GMS*-treated wine, potentially contributing to a rich fruity and floral aroma and increasing the complexity of the wine’s fragrance.

#### 3.2.5. Sensory Evaluation

**Figure 6:**
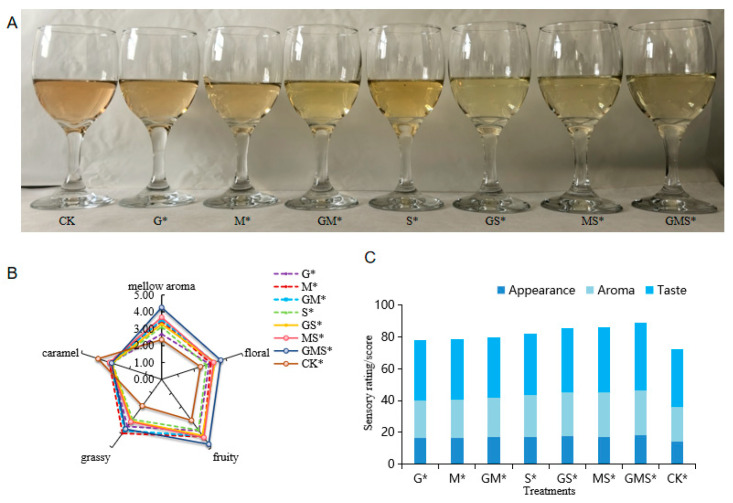
(**A**–**C**) Sensory evaluation of semi-sweet wine treated with various combinations of antioxidants. (**A**) Wine samples under different treatments; (**B**) the distinct aroma characteristics of the wine samples under various treatments; (**C**) the overall taste description scores for the wine samples.

Sensory evaluation of the semi-sweet wines treated with different combinations of antioxidants was conducted. As shown in Figure 6A, among the wine samples under different treatments, the colors of GS, MS and GMS are lighter. This indicates that the degree of color oxidation is lower in these samples. As shown in Figure 6B, wines treated with different methods exhibited distinct aroma profiles. Figure 6C presents the overall taste description scores, where the CK* sample showed evidence of oxidation, with an amber color, caramel aroma, high acidity, and poor balance, resulting in the lowest score. The G*, M*, and GM* treatments resulted in wine samples with lighter colors than CK, with a lighter oxidation odor, elegant and complex fruity aromas, and well-preserved acidity. The wine samples treated with 20 mg/L SO_2_ had a light amber color. When SO_2_ was added in combination with GSH and Man, the wine samples presented a light golden yellow color with mild oxidation. The wines without SO_2_ treatment showed stronger plant-like aroma characteristics, while the addition of natural antioxidants significantly increased the floral and berry aromas of the wine samples. The GS*-treated wine was light golden yellow with a fresh and crisp taste; the MS*-treated wine had a full body and rich flavor. The wine samples treated with GMS* exhibited rich fruity and floral aromas. This may be related to the high content of ester aroma compounds detected in the GMS* treatment. The GMS* treatment, which involved the action of three antioxidants, scored the highest at 88.3 points, standing out for its fresh and refreshing taste, full body, complex and balanced aroma.

## 4. Conclusions

This study focused on the impact of GSH, Man, VC, COS and SO_2_ on the quality of the ‘Petit Manseng’ semi-sweet wine. The results showed that all four natural antioxidants could enhance the antioxidant capacity of the wine, with VC being particularly effective in scavenging oxygen and free radicals. GSH and Man stood out in delaying color oxidation and optimizing the quality of tannins, providing a new perspective for wine antioxidant research. This study systematically analyzed the comprehensive effects of combined treatments of multiple antioxidants on wine quality. The research indicated that the GSH treatment increased the amino acid content, endowing the wine with complex and rich fruity and floral aromas. The Man treatment increased the content of monophenolic substances, enriching the aromatic components of the wine. The GMS* treatment yielded the best results: the concentration of SO_2_ was reduced by 33.3%, and the wine achieved the highest sensory scores. It exhibited a light yellow color, a fresh and refreshing taste, a full body, and a complex and balanced aroma, meeting consumers’ dual demands for health and high-quality wine.

## 5. Patents

One international patent has been granted for the content of this study, with the patent name “ANTIOXIDANT METHOD FOR LOW-SULFUR SEMI-SWEET WINE”.

## Figures and Tables

**Table 1:** Physical and chemical indicators of semi-sweet wine.

Treatment	Alcohol Content (% vol)	pH	Residual Sugar (g/L)	Total Acidity (g/L)	Dry Extract (g/L)	Free Sulfur (mg/L)	Bound Sulfur (mg/L)
Single treatment	13.0	3.1	17.7	5.3	27.7	2.2	12.0
Combined treatment	12.0	3.2	17.5	5.1	27.3	1.7	13.2

**Table 2:** Different methods of adding antioxidants to the wine samples.

Treatment Code	Treatment Method
CK	Distilled water blank
G-1	10 mg/L GSH
G-2	20 mg/L GSH
G-3	30 mg/L GSH
M-1	10 mg/L Man
M-2	20 mg/L Man
M-3	30 mg/L Man
V-1	50 mg/L VC
V-2	100 mg/L VC
V-3	200 mg/L VC
C-1	50 mg/L COS
C-2	100 mg/L COS
C-3	200 mg/L COS
S-1	10 mg/L SO_2_
S-2	20 mg/L SO_2_
S-3	30 mg/L SO_2_
CK*	Distilled water blank
G*	10 mg/L GSH
M*	10 mg/L Man
GM*	10 mg/L GSH and 10 mg/L Man
S*	20 mg/L SO_2_
GS*	10 mg/L GSH and 20 mg/L SO_2_
MS*	10 mg/L Man and 20 mg/L SO_2_
GMS*	5 mg/L GSH, 5 mg/L Man and 20 mg/L SO_2_

**Table 4:** Differences in polyphenol content in semi-sweet wine treated with various combinations of antioxidants.

Chemical Substance	Concentration (µg/L)							
	CK*	G*	M*	GM*	S*	GS*	MS*	GMS*
Caffeic acid	1217.5 ± 50.8 ^f^	1594.6 ± 281.9 ^d^	1500.3 ± 129.7 ^d^	1479.2 ± 47.1 ^d^	3443.3 ± 543.9 ^c^	4058.2 ± 197.5 ^ab^	3889.2 ± 214.1 ^b^	4265.8 ± 111.3 ^a^
Gallic acid	356.0 ± 26.3 ^f^	394.2 ± 8.4 ^f^	1078.0 ± 102.1 ^e^	2115.8 ± 22.4 ^d^	2527.4 ± 457.2 ^c^	3018.9 ± 283.6 ^a^	2699.2 ± 230.7 ^b^	3089.9 ± 289.7 ^a^
Coumaric acid	278.1 ± 9.1 ^b^	278.5 ± 19.1 ^b^	278.0 ± 10.2 ^b^	272.8 ± 10.7 ^b^	416.3 ± 36.2 ^a^	427.9 ± 28.6 ^a^	424.2 ± 24.2 ^a^	459.1 ± 31.1 ^a^
Gallocatechin	213.3 ± 10.3 ^d^	209.4 ± 21.9 ^d^	372.1 ± 18.3 ^c^	411.3 ± 31.2 ^b^	368.4 ± 39.4 ^c^	427.7 ± 42.1 ^b^	543.8 ± 44.3 ^a^	567.4 ± 36.4 ^a^
Vanillic acid	177.5 ± 13.2 ^a^	179.1 ± 5.3 ^a^	185.9 ± 6.3 ^a^	180.3 ± 3.6 ^a^	189.3 ± 4.3 ^a^	179.9 ± 9.1 ^a^	185.8 ± 2.4 ^a^	180.3 ± 10.3 ^a^
Ferulic acid	158.8 ± 2.8 ^d^	171.4 ± 11.2 ^d^	167.3 ± 8.4 ^d^	167.7 ± 3.2 ^d^	229.6 ± 15.2 ^c^	231.1 ± 7.1 ^bc^	236.5 ± 7.1 ^ab^	247.1 ± 5.9 ^a^
Syringic acid	150.1 ± 2.9 ^b^	149.1 ± 3.4 ^b^	150.3 ± 3.1 ^b^	151.9 ± 1.2 ^b^	170.9 ± 5.1 ^a^	167.6 ± 3.9 ^a^	170.1 ± 5.9 ^a^	171.7 ± 2.2 ^a^
Salicylic acid	147.1 ± 6.2 ^b^	153.5 ± 1.7 ^ab^	150.4 ± 6.3 ^ab^	151.7 ± 6.4 ^ab^	160.4 ± 2.2 ^a^	155.1 ± 5.1 ^ab^	160.6 ± 0.6 ^a^	161.4 ± 1.5 ^a^
Catechin	117.7 ± 14.5 ^d^	147.6 ± 32.2 ^c^	128.8 ± 10.5 ^cd^	136.6 ± 12.9 ^c^	531.6 ± 214.5 ^b^	672.2 ± 69.6 ^a^	558.4 ± 41.1 ^b^	663.5 ± 58.4 ^a^
Epicatechin	108.1 ± 10.2 ^f^	166.2 ± 40.1 ^d^	137.4 ± 13.6 ^d^	145.4 ± 8.1 ^d^	570.4 ± 0.6 ^c^	730.1 ± 75.4 ^a^	610.6 ± 51.1 ^b^	738.2 ± 52.6 ^a^
Resveratrol 3-O-glucoside	103.4 ± 1.8 ^c^	104.4 ± 11.7 ^c^	107.5 ± 5.2 ^c^	105.1 ± 2.7 ^c^	153.2 ± 9.9 ^a^	133.7 ± 13.4 ^b^	127.9 ± 9.1 ^b^	140.5 ± 17.1 ^ab^
Quercetin-3-O-rhamnoside	71.3 ± 0.2 ^b^	71.2 ± 0.4 ^b^	71.1 ± 0.5 ^b^	71.2 ± 0.3 ^b^	72.2 ± 0.9 ^a^	69.7 ± 0.5 ^c^	69.1 ± 0.6 ^c^	70.1 ± 0.4 ^bc^
Epigallocatechin	36.2 ± 3.7 ^d^	42.8 ± 9.5 ^cd^	45.3 ± 1.6 ^c^	39.7 ± 6.1 ^cd^	72.1 ± 4.5 ^b^	122.9 ± 16.3 ^a^	74.7 ± 15.7 ^b^	119.1 ± 12.6 ^a^
Petunidin-3-O-glucoside	32.1 ± 0.5 ^a^	32.1 ± 1.1 ^a^	32.5 ± 0.2 ^a^	31.9 ± 0.6 ^a^	32.3 ± 0.6 ^a^	32.3 ± 0.7 ^a^	32.4 ± 0.4 ^a^	31.6 ± 0.3 ^a^
Procyanidin B1	17.9 ± 0.6 ^c^	22.1 ± 0.6 ^c^	18.7 ± 2.1 ^c^	19.8 ± 2.2 ^c^	142.7 ± 10.2 ^ab^	149.0 ± 10.7 ^a^	97.4 ± 7.1 ^b^	146.9 ± 26.5 ^ab^
Resveratrol	13.5 ± 0.1 ^b^	13.7 ± 0.1 ^a^	13.5 ± 0.1 ^b^	13.7 ± 0.1 ^a^	13.6 ± 0.1 ^a^	13.8 ± 0.1 ^a^	13.5 ± 0.1 ^a^	13.7 ± 0.4 ^ab^
Epicatechin gallate	6.1 ± 0.3 ^a^	4.9 ± 0.2 ^b^	4.6 ± 0.1 ^b^	4.6 ± 0.1 ^b^	5.8 ± 1.2 ^ab^	4.7 ± 0.2 ^b^	4.6 ± 0.1 ^b^	4.3 ± 0.1 ^c^
Isorhamnetin-3-O-glucoside	1.2 ± 0.2 ^a^	1.1 ± 0.1 ^a^	1.1 ± 0.1 ^a^	1.1 ± 0.1 ^a^	1.1 ± 0.1 ^a^	1.1 ± 0.1 ^a^	1.1 ± 0.1 ^a^	1.1 ± 0.1 ^a^

**Note**: In the table, the letters ^a–f^ are used to indicate statistical differences among different treatment groups at the significance level where *p* ≤ 0.05.

**Table 5:** Differences in amino acid content in semi-sweet wine under the combined effects of various antioxidants.

Chemical Substance	Concentration (mg/L)							
	CK*	G*	M*	GM*	S*	GS*	MS*	GMS*
Prolin	2691.1 ± 338.5 ^ab^	2353.1 ± 287.1 ^b^	2671.1 ± 299.1 ^ab^	2444.2 ± 228.4 ^b^	2558.6 ± 109.2 ^ab^	2547.5 ± 150.8 ^b^	2733.6 ± 285.7 ^ab^	2777.3 ± 222.1 ^a^
Arginin	110.3 ± 3.3 ^b^	107.8 ± 4.9 ^b^	112.2 ± 3.4 ^ab^	110.5 ± 3.4 ^b^	111.3 ± 2.8 ^b^	114.4 ± 2.9 ^ab^	116.5 ± 0.6 ^a^	112.1 ± 3.5 ^ab^
G-aminobutyric acid	81.1 ± 2.4 ^a^	80.4 ± 3.2 ^a^	84.7 ± 2.5 ^a^	84.2 ± 2.5 ^a^	84.5 ± 1.8 ^a^	82.1 ± 2.1 ^a^	83.6 ± 0.8 ^a^	81.1 ± 2.4 ^a^
Alanin	39.6 ± 1.3 ^a^	39.4 ± 1.6 ^a^	41.6 ± 1.2 ^a^	41.3 ± 1.2 ^a^	41.4 ± 0.9 ^a^	40.3 ± 1.0 ^a^	41.1 ± 1.2 ^a^	40.1 ± 1.4 ^a^
Valin	29.4 ± 1.1 ^ab^	29.2 ± 1.1 ^ab^	30.8 ± 0.8 ^a^	30.2 ± 0.8 ^a^	30.5 ± 0.6 ^a^	29.3 ± 0.9 ^ab^	29.8 ± 0.3 ^ab^	28.6 ± 0.9 ^b^
Glutamic acid	27.1 ± 0.8 ^a^	27.1 ± 1.1 ^a^	28.2 ± 0.9 ^a^	28.1 ± 0.9 ^a^	28.2 ± 0.6 ^a^	28.2 ± 0.7 ^a^	28.8 ± 0.2 ^a^	27.9 ± 0.8 ^a^
β-Alanin	23.2 ± 0.6 ^a^	23.1 ± 0.8 ^a^	24.3 ± 0.7 ^a^	24.2 ± 0.7 ^a^	24.2 ± 0.5 ^a^	23.8 ± 0.6 ^a^	24.3 ± 0.7 ^a^	23.6 ± 0.6 ^a^
Lysin	22.3 ± 1.1 ^a^	22.3 ± 1.4 ^a^	23.7 ± 0.3 ^a^	23.9 ± 1.3 ^a^	23.8 ± 0.4 ^a^	23.2 ± 0.8 ^a^	23.7 ± 0.8 ^a^	22.8 ± 0.7 ^a^
Ornithin	14.4 ± 0.3 ^ab^	14.1 ± 0.7 ^b^	14.7 ± 0.4 ^ab^	14.9 ± 0.5 ^ab^	14.8 ± 0.1 ^ab^	14.8 ± 0.6 ^ab^	15.2 ± 0.6 ^a^	14.5 ± 0.2 ^ab^
Aspartic acid	14.2 ± 1.5 ^a^	14.4 ± 1.4 ^a^	15.1 ± 1.2 ^a^	13.6 ± 1.2 ^a^	14.3 ± 2.1 ^a^	15.1 ± 0.9 ^a^	15.7 ± 1.4 ^a^	14.4 ± 1.3 ^a^
Leucin	13.8 ± 0.4 ^a^	13.8 ± 0.5 ^a^	14.6 ± 0.4 ^a^	14.6 ± 0.4 ^a^	14.6 ± 0.3 ^a^	14.1 ± 0.3 ^a^	14.4 ± 0.1 ^a^	14.1 ± 0.4 ^a^
Asparagin	13.2 ± 0.3 ^a^	13.2 ± 0.4 ^a^	13.8 ± 0.4 ^a^	13.8 ± 0.4 ^a^	13.8 ± 0.3 ^a^	13.6 ± 0.3 ^a^	13.9 ± 0.1 ^a^	13.6 ± 0.4 ^a^
Glycin	12.1 ± 0.1 ^b^	12.1 ± 0.3 ^b^	12.6 ± 0.4 ^ab^	12.6 ± 0.4 ^ab^	12.6 ± 0.3 ^a^	12.6 ± 0.3 ^a^	12.9 ± 0.1 ^a^	12.5 ± 0.3 ^ab^
Histidin	11.1 ± 0.5 ^b^	11.1 ± 0.6 ^b^	11.3 ± 0.4 ^b^	11.3 ± 0.4 ^b^	11.3 ± 0.3 ^ab^	13.3 ± 0.3 ^a^	12.1 ± 0.8 ^ab^	12.1 ± 0.4 ^ab^
Glutamin	10.4 ± 0.3 ^c^	10.4 ± 0.4 ^c^	11.1 ± 0.3 ^b^	11.1 ± 0.3 ^b^	11.1 ± 0.2 ^b^	12.1 ± 0.2 ^a^	10.6 ± 0.3 ^c^	10.9 ± 0.3 ^c^
Phenylalanin	9.9 ± 0.3 ^a^	10.0 ± 0.4 ^a^	10.6 ± 0.3 ^a^	10.5 ± 0.2 ^a^	10.5 ± 0.2 ^a^	10.2 ± 0.1 ^a^	10.5 ± 0.2 ^a^	10.2 ± 0.2 ^a^
Tyrosin	9.1 ± 0.3 ^a^	9.1 ± 0.4 ^a^	9.6 ± 0.3 ^a^	9.5 ± 0.3 ^a^	9.5 ± 0.2 ^a^	9.4 ± 0.1 ^a^	9.6 ± 0.3 ^a^	9.3 ± 0.2 ^a^
Serin	6.6 ± 0.2 ^b^	6.7 ± 0.2 ^b^	7.1 ± 0.1 ^a^	7.0 ± 0.1 ^a^	7.1 ± 0.1 ^a^	6.9 ± 0.1 ^a^	7.2 ± 0.1 ^a^	7.0 ± 0.1 ^a^
Methionin	5.3 ± 1.2 ^b^	6.1 ± 1.2 ^ab^	6.5 ± 0.4 ^a^	4.6 ± 0.4 ^b^	5.6 ± 1.4 ^ab^	4.7 ± 1.6 ^b^	5.2 ± 1.7 ^ab^	4.2 ± 1.2 ^b^
Threonin	3.1 ± 0.3 ^b^	3.4 ± 0.3 ^ab^	3.4 ± 0.1 ^ab^	3.5 ± 0.1 ^a^	3.4 ± 0.1 ^ab^	3.4 ± 0.1 ^ab^	3.4 ± 0.1 ^ab^	3.4 ± 0.1 ^ab^
Isoleucin	2.6 ± 0.1 ^a^	2.7 ± 0.1 ^a^	2.8 ± 0.1 ^a^	2.8 ± 0.1 ^a^	2.8 ± 0.1 ^a^	2.7 ± 0.1 ^a^	2.8 ± 0.1 ^a^	2.8 ± 0.1 ^a^

**Note:** In the table, the letters ^a^, ^b^, ^c^ are used to indicate statistical differences among different treatment groups at the significance level where *p* ≤ 0.05.

**Table 6:** Differences in the content of volatile compounds in semi-sweet wines under the combined effects of different antioxidants.

Retention Time	Volatile Chemical Substances	Concentration (µg/L)							
		CK*	G*	M*	GM*	S*	GS*	MS*	GMS*
3.93	Isoamyl alcohol	1390.4 ± 214.2 ^ab^	1026.5 ± 109.2 ^d^	1388.2 ± 108.9 ^b^	1453.3 ± 217.3 ^a^	1346.2 ± 37.9 ^b^	1488.3 ± 27.4 ^a^	1395.1 ± 79.2 ^ab^	1227 ± 73.2 ^c^
5.5	2,3-Butanediol	35.4 ± 8.2 ^a^	20.1 ± 2.1 ^b^	18.5 ± 2.8 ^bc^	21.4 ± 3.8 ^b^	13.5 ± 1.1 ^cd^	14.7 ± 2.^c^	12.8 ± 1.2 ^d^	15.3 ± 1.2 ^c^
5.75	n-Pentanol	31.4 ± 2.3 ^d^	54.8 ± 2.9 ^c^	62.4 ± 1.3 ^b^	60.5 ± 3.8 ^b^	72.5 ± 3.1 ^a^	68.4 ± 1.5 ^b^	75.2 ± 2.7 ^a^	69.5 ± 0.8 ^b^
7.75	n-Hexanol	194.6 ± 13.9 ^e^	264.8 ± 14.3 ^d^	276.8 ± 8.4 ^d^	288.4 ± 15.2 ^d^	425.9 ± 13.7 ^a^	395.1 ± 9.3 ^c^	468.5 ± 21.3 ^a^	421.5 ± 11.9 ^b^
14.43	Cyclohexanol	2.6 ± 1.8 ^e^	10.6 ± 1.8 ^d^	14.2 ± 1.8 ^c^	12.9 ± 2.1 ^d^	12.4 ± 1.1 ^d^	18.1 ± 1.8 ^b^	22.6 ± 2.3 ^a^	17.8 ± 2.1 ^b^
16.89	n-Octanol	16.8 ± 1.3 ^e^	18.6 ± 2.3 ^de^	20.5 ± 1.1 ^d^	21.5 ± 0.4 ^d^	32.7 ± 2.9 ^b^	28.5 ± 1.7 ^bc^	36.8 ± 2.3 ^a^	30.7 ± 2.8 ^bc^
18.85	Phenylethyl alcohol	91.8 ± 3.4 ^a^	66.4 ± 3.2 ^b^	45.6 ± 2.1 ^d^	58.2 ± 3.1 ^c^	58.3 ± 2.1 ^c^	34.3 ± 2.1 ^e^	58.5 ± 2.9 ^c^	44.6 ± 2.1 ^d^
26.37	n-Decanol	7.4 ± 0.3 ^d^	24.4 ± 1.2 ^bc^	24.2 ± 2.1 ^bc^	22.3 ± 1.1 ^c^	26.3 ± 2.1 ^b^	31.5 ± 1.9 ^a^	31.9 ± 0.3 ^a^	32.5 ± 1.2 ^a^
2.28	Ethyl acetate	318.2 ± 1.3 ^e^	420.7 ± 5.9 ^d^	518.3 ± 21.8 ^c^	687.4 ± 15.3 ^a^	574.1 ± 30.2 ^b^	308.1 ± 15.3 ^e^	558.4 ± 12.4 ^b^	578 ± 3.8 ^b^
3.43	Ethyl propionate	32.8 ± 2.8 ^d^	50.6 ± 5.9 ^c^	48.7 ± 3.8 ^c^	52.5 ± 3.8 ^c^	67.6 ± 3.1 ^b^	72.8 ± 2.1 ^a^	71.7 ± 1.4 ^a^	66.8 ± 2.2 ^b^
4.62	Isobutyl acetate	20.7 ± 2.3 ^e^	12.5 ± 3.2 ^f^	50.2 ± 2.7 ^c^	38.9 ± 2.1 ^d^	65.1 ± 3.2 ^b^	56.4 ± 3.1 ^c^	70.7 ± 2.1 ^a^	65.2 ± 2.1 ^b^
5.32	Ethyl butyrate	120.9 ± 13.8 ^e^	233.5 ± 15.9 ^c^	239.5 ± 11.9 ^c^	155.4 ± 7.3 ^d^	354.2 ± 3.9 ^b^	359.2 ± 3.5 ^b^	472.1 ± 15.2 ^a^	350.1 ± 8.3 ^b^
7.01	Isoamyl valerate	2.6 ± 0.3 ^bc^	1.9 ± 0.1 ^d^	2.2 ± 0.2 ^c^	2.3 ± 0.1 ^c^	3.2 ± 0.1 ^c^	3.7 ± 0.2 ^a^	3.6 ± 1.1 ^a^	3.3 ± 1.3 ^b^
7.9	Isoamyl acetate	1529.2 ± 34.8 ^e^	1811.5 ± 104.2 ^d^	2210.5 ± 207.2 ^c^	1880.7 ± 128.8 ^d^	2253.6 ± 117.3 ^c^	2524.2 ± 47.1 ^b^	3297.3 ± 142.8 ^a^	3352.1 ± 231.2 ^a^
9.35	Amyl acetate	0.8 ± 0.2 ^d^	0.8 ± 0.3 ^d^	1.2 ± 0.1 ^d^	1.1 ± 0.2 ^d^	3.0 ± 0.2 ^a^	1.8 ± 0.1 ^c^	2.3 ± 0.2 ^b^	1.8 ± 0.2 ^c^
12.35	3-Methyl amyl acetate	5.6 ± 0.1 ^b^	4.1 ± 0.1 ^e^	4.6 ± 0.1 ^d^	5.2 ± 0.1 ^c^	5.3 ± 0.1 ^bc^	6.6 ± 0.15 ^a^	5.7 ± 0.2 ^b^	5.6 ± 0.1 ^b^
13.83	Hexyl acetate	555.1 ± 34.1 ^d^	928.7 ± 104.2 ^c^	1071.2 ± 73.2 ^b^	900.1 ± 25.3 ^c^	1335.8 ± 74.1 ^a^	1334.6 ± 109.0 ^a^	1276.6 ± 134.3 ^a^	1225.6 ± 93.2 ^a^
15.89	Isoamyl butyrate	2.5 ± 0.1 ^b^	1.8 ± 0.1 ^c^	2.2 ± 0.2 ^c^	2.6 ± 0.1 ^b^	3.2 ± 0.1 ^ab^	3.9 ± 0.1 ^a^	3.7 ± 0.1 ^a^	3.6 ± 0.2 ^a^
17.91	Ethyl heptanoate	1.2 ± 0.1 ^a^	0.7 ± 0.1 ^c^	0.9 ± 0.1 ^b^	1.1 ± 0.1 ^b^	0.9 ± 0.1 ^b^	1.3 ± 0.1 ^a^	0.9 ± 0.1 ^b^	0.8 ± 0.1 ^bc^
20.44	Isobutyl hexanoate	2.2 ± 0.1 ^e^	3.2 ± 0.1 ^d^	3.6 ± 0.1 ^c^	3.9 ± 0.1 ^c^	4.4 ± 0.1 ^b^	5.7 ± 0.1 ^a^	4.4 ± 0.1 ^b^	4.4 ± 0.1 ^b^
22.82	Ethyl octanoate	5682.3 ± 538.9 g	7043.2 ± 338.2 ^f^	7603.1 ± 834.1 ^f^	9107.5 ± 593.1 ^e^	17,839.1 ± 398.3 ^c^	199,028.3 ± 903.2 ^a^	13,961.2 ± 739.3 ^d^	157,220 ± 1098.3 ^b^
25.11	Isoamyl hexanoate	51.8 ± 5.9 ^c^	80.1 ± 5.9 ^d^	90.2 ± 9.3 ^b^	81.7 ± 3.9 ^c^	99.4 ± 4.9 ^b^	111.1 ± 3.8 ^a^	109.3 ± 7.9 ^a^	108.9 ± 6.1 ^ab^
25.38	Phenethyl acetate	25.2 ± 2.9 ^e^	42.1 ± 3.3 ^d^	25.1 ± 3.3 ^e^	28.6 ± 3.9 ^e^	80.2 ± 2.9 ^a^	55.9 ± 3.9 ^c^	74.7 ± 2.7 ^b^	75.5 ± 3.2 ^b^
27.04	Propyl octanoate	2.7 ± 0.2 ^c^	3.5 ± 0.1 ^b^	3.8 ± 0.2 ^b^	3.9 ± 0.7 ^b^	4.6 ± 0.2 ^a^	4.7 ± 0.2 ^a^	5.2 ± 0.3 ^a^	4.5 ± 0.3 ^ab^
27.21	Ethyl nonanoate	6.3 ± 0.3 ^e^	10.3 ± 0.7 ^c^	8.6 ± 1.1 ^d^	1.9 ± 0.1 ^f^	13.5 ± 1.2 ^b^	13.2 ± 0.3 ^b^	15.2 ± 0.9 ^a^	12.8 ± 0.8 ^b^
28.46	Methyl decanoate	5.6 ± 0.9 ^e^	10.4 ± 0.2 ^c^	8.2 ± 0.3 ^d^	10.7 ± 0.4 ^c^	14.6 ± 0.2 ^a^	13.1 ± 0.3 ^ab^	12.2 ± 0.2 ^b^	13.7 ± 0.7 ^a^
29.96	Ethyl 9-decenoate	264.5 ± 23.9 ^f^	585.1 ± 38.1 ^e^	650.1 ± 76.2 ^d^	655.3 ± 33.7 ^d^	764.9 ± 23.9 ^c^	861.7 ± 29.3 ^b^	994.6 ± 39.2 ^a^	813.8 ± 34.9 ^b^
31.72	Ethyl decanoate	12,272.1 ± 428.3 ^d^	15,043.2 ± 38.9 ^b^	15,275.2 ± 870.6 ^b^	15,092.3 ± 637.3 ^b^	16,425.8 ± 398.2 ^a^	15,443.9 ± 329.2 ^b^	14,275.5 ± 378.1 ^c^	14,271.3 ± 398.2 ^c^
33.64	Isoamyl octanoate	61.6 ± 12.9 ^a^	15.6 ± 2.9 ^b^	15.6 ± 0.3 ^c^	16.4 ± 1.9 ^b^	13.4 ± 1.2 ^b^	14.6 ± 0.8 ^bc^	11.1 ± 0.3 ^d^	11.2 ± 1.2 ^d^
35.15	Ethyl 9-hexadecenoate	6.4 ± 0.4 ^c^	11.21 ± 0.35 ^a^	10.6 ± 0.7 ^b^	8.2 ± 0.3 ^b^	12.7 ± 1.1 ^a^	12.3 ± 0.3 ^a^	12.0 ± 0.7 ^a^	12.0 ± 0.5 ^a^
35.37	n-Propyl decanoate	0.4 ± 0.1 ^c^	0.6 ± 0.1 ^a^	0.6 ± 0.1 ^a^	0.7 ± 0.1 ^a^	0.7 ± 0.1 ^a^	0.5 ± 0.1 ^ab^	0.7 ± 0.1 ^a^	0.8 ± 0.1 ^a^
37.29	Isobutyl decanoate	0.2 ± 0.1 ^d^	1.5 ± 0.1 ^c^	1.9 ± 0.1 ^b^	2.4 ± 0.2 ^a^	1.9 ± 0.1 ^b^	1.8 ± 0.1 ^b^	1.3 ± 0.1 ^c^	1.3 ± 0.1 ^c^
37.54	Ethyl myristate	0.5 ± 0.1 ^d^	1.3 ± 0.1 ^cd^	2.2 ± 0.1 ^b^	2.8 ± 0.1 ^a^	3.1 ± 0.1 ^a^	2.6 ± 0.1 ^ab^	1.7 ± 0.1 ^c^	0.9 ± 0.1 ^d^
37.88	Ethyl palmitate	1.1 ± 0.1 ^c^	3.7 ± 0.1 ^a^	3.5 ± 0.2 ^a^	3.6 ± 0.2 ^a^	3.1 ± 0.3 ^ab^	3.5 ± 0.3 ^a^	2.8 ± 0.1 ^b^	2.5 ± 0.3 ^b^
38.91	Ethyl laurate	1103.1 ± 74.2 ^c^	1525.6 ± 105.3 ^a^	1426.7 ± 78.1 ^a^	1516.1 ± 78.1 ^a^	1417.4 ± 78.2 ^ab^	1389.4 ± 107.2 ^b^	1298.7 ± 78.8 ^b^	1435.2 ± 36.9 ^a^
40.52	3-Methylbutyl decanoate	6.9 ± 02 ^b^	4.6 ± 0.6 ^c^	6.3 ± 0.2 ^b^	9.1 ± 0.2 ^a^	7.1 ± 0.3 ^b^	6.7 ± 0.1 ^b^	4.6 ± 0.7 ^c^	4.9 ± 0.3 ^c^
24.56	Octanoic acid	6.2 ± 0.2 ^a^	4.7 ± 0.3 ^b^	3.7 ± 0.1 ^bc^	2.2 ± 0.1 ^cd^	2.6 ± 0.1 ^c^	2.3 ± 0.1 ^c^	2.9 ± 0.1 ^c^	1.8 ± 0.1 ^d^
32.64	Decanoic acid	5.4 ± 0.1 ^a^	3.8 ± 0.1 ^b^	3.2 ± 0.1 ^b^	2.1 ± 0.3 ^bc^	1.8 ± 0.1 ^c^	3.1 ± 0.2 ^b^	2.9 ± 0.1 ^b^	2.7 ± 0.1 ^b^
1.68	Acetaldehyde	71.4 ± 2.9 ^a^	54.5 ± 3.9 ^b^	46.4 ± 5.1 ^b^	50.8 ± 2.3 ^b^	40.8 ± 5.9 ^bc^	35.2 ± 4.2 ^c^	32.6 ± 7.24 ^c^	28.1 ± 2.2 ^d^
3.64	Acetal	122.1 ± 11.8 ^a^	45.7 ± 4.3 ^c^	74.7 ± 4.2 ^b^	75.2 ± 2.9 ^b^	51.6 ± 5.3 ^c^	44.3 ± 3.2 ^c^	51.1 ± 5.2 ^c^	40.2 ± 5.2 ^c^
19.18	Benzaldehyde	15.5 ± 2.1 ^a^	10.6 ± 1.2 ^b^	9.6 ± 1.1 ^b^	12.6 ± 2.3 ^a^	5.4 ± 0.3 ^c^	4.2 ± 0.7 ^c^	3.5 ± 0.3 ^c^	4.8 ± 0.2 ^c^
13.05	Linalool	10.3 ± 3.2 ^a^	2.2 ± 0.1 ^c^	1.8 ± 0.3 ^a^	2.2 ± 0.7 ^b^	2.1 ± 0.2 ^b^	1.7 ± 0.1 ^b^	2.2 ± 0.3 ^b^	1.8 ± 0.1 ^b^
30.65	Damascenone	32.1 ± 1.2 ^a^	16.5 ± 1.1 ^b^	12.2 ± 1.2 ^b^	15.3 ± 2.1 ^b^	14.4 ± 0.2 ^b^	12.8 ± 1.2 ^c^	10.7 ± 0.3 ^c^	11.5 ± 1.2 ^c^
33.64	2,4-Di-tert-butylphenol	3.9 ± 0.1 ^a^	1.8 ± 0.1 ^b^	1.6 ± 0.1 ^b^	1.1 ± 0.1 ^c^	1.1 ± 0.1 ^c^	0.5 ± 0.1 ^d^	0.7 ± 0.1 ^c^	0.8 ± 0.1 ^c^

**Note:** In the table, the letters ^a–f^ are used to indicate statistical differences among different treatment groups at the significance level where *p* ≤ 0.05.

## Data Availability

The original contributions presented in the study are included in the article, further inquiries can be directed to the corresponding authors.

## Abbreviations

The following abbreviations are used in this manuscript:

GSH	Glutathione
Man	Mannan
VC	Vitamin C
COS	Chitosan oligosaccharide
PCA	Principal component analysis
